# “Novice” Noticed

**Published:** 1858-03

**Authors:** 


### "NOVICE" NOTICED.

Among the writings of an eminent author of the last century, I find the following: "There are wild asses in South America. They have three properties which bear a moral application. 1. Though exceedingly swift, fierce, and untractable, after carrying the first load, their celerity leaves them, their dangerous ferocity is lost, and they contract the stupid look and dullness of the assine species: one of them becomes like another ass. 2. If that more noble animal, a horse, happens to stray into the place where they feed, they all fall upon him; and without giving him the liberty of flying from them, they bite and kick him, till they leave him dead on the spot. 3. They are very troublesome neighbors, making a most horrid noise; for, whenever one or two of them *begin to bray*, they are answered in the same vociferous manner by all within reach of the sound, which is greatly increased and prolonged by the representations of the valleys and breaches of the mountains."

I have cited this account of the wild asses of South America, on account of the appearance in a recent number of the "*Dental Register of the West*," of an article over the very appropriate signature of "Novice," the object of which is to criticise an article on *Cheoplasty and its results*, which appeared in the October number of the American Journal of Dental Science. In the article referred to, I deemed it proper that my views should be based upon *facts*. And facts to be entitled to respectful consideration should not be referred to in *general* terms only, but should be definitely stated, and accompanied by sufficient vouchers. Consequently, I furnished two letters confirmatory of the opinion there advanced. One of the letters was from the pen of a gentleman of the medical faculty, of very high scientific attainments, and to whom the public have often been indebted for valuable contributions on philosophical subjects. The other letter was from a well known and highly respected clergyman of this State, and which bears internal evidence that he is not altogether a "*novice*" in the matter on which he writes. The testimony in favor of Cheoplasty, contained in these letters, is just such as the occasion required. It combines acknowledged scientific capacity—ample opportunities for judging—sufficient time to test the correctness of opinions—and all requisite moral qualifications to give it weight. It is accompanied, moreover, by the real names of the parties testifying, and which, in the minds of those who know them, are never associated with "humbug" or duplicity.

How is it sought to evade the force of that testimony? Is it by any positive counteracting evidence? Is it by adducing any well authenticated facts, accompanied by names of witnesses as competent and as responsible as those advanced by me?

Nothing of the kind. A reckless scribbler, skulking under cover of an anonymous signature, offers, with the very perfection of impudence, and with unmistakable indications of gross ignorance, what? Simply his irresponsible *ipse dixit.* A "*Novice*," and obviously a novice, indeed, coolly asks an intelligent profession to reject testimony as positive, as intelligent, as credible, as far above suspicion of corrupt influences, as any that ever decided questions touching the welfare, the reputation, the property, or even life of men: and to do this, forsooth, because *he* without giving his *own name*, (which is, perhaps, the wisest part of his performance,) or the name of any man, woman, or child, tells us that a nicely finished piece of Dr. Blandy's work, placed on a shelf in any laboratory, during ten days, will exhibit evidences of corrosion similar to that of an old spoon in a neglected scullery. This, with the general tone of gnarling and sneering, which this veritable "Novice" has adopted; and his dull stupidity in supposing that an intelligent profession can be hoodwinked by the statements of an anonymous writer, though familiar with neglected sculleries, in opposition to well certified facts, indicate that our scribbler bears a strong resemblance to the animals we have alluded to in the beginning of this article.

Seemingly impressed, however, with a sense of the necessity of *some show* of facts, "Novice" tells us that "a very clever member of our profession, in St. Louis, made a pilgrimage to Baltimore, where, treated like a veritable devotee to Cheoplasty, he was favorably impressed with it; and with very limited opportunities to watch practical results, returning to his home in time to be present at a meeting of the Western Dental Society, and was one of a committee which prepared a favorable report which was adopted. But the result of a sober second thought, aided by ample experience, is, that Cheoplasty is not used by any one in St. Louis (so we understand) or Cincinnati."

If this be worth anything, its value must be tested by a fuller statement than this "novice" has ventured to give us.

It should have included some account of that "sober second thought," and should have furnished us with some facts, upon which that "ample experience" was based, and which has resulted in the exclusion of cheoplasty from St. Louis and Cincinnati. In the total absence of all specifications—names and other circumstances essential to the value of testimony; and in view of the fact that it is offered in an anonymous article, what assurance have we that the whole story has not been fabricated for the occasion. But, *if true*, what does it amount to? Simply this: that these "clever ones" of St. Louis and Cincinnati, have been less *fortunate* in the use of a system, which, in the hands of gentlemen, both here and elsewhere, of tried skill and integrity, have obtained for them results so decidedly satisfactory, as to cause its adoption to the entire exclusion of all other modes of inserting artificial teeth.

I know not what "Diana of the Ephesians" "Novice" is dedicated to, but it is evident that he suspects that his "craft is in danger." Let the profession in the West allow themselves to be blinded to facts, and deprive their fellow beings of the benefits connected with a most valuable invention, simply by the unsupported *ipse dixit* of "Novice.' Let prejudices, selfishness, and bigotry frown upon the developments of science, and plodding on, according to the old system which experience has proven imperfect—continue to spurn the discoveries which reward patient toil and study, and we may apply to the noble West the lines of Collins in his Oriental Eclogues:

> "Here where no springs in murmurs break away,
> Or moss-crowned fountains mitigate the day,
> In vain you hope the dear delight to know,
> Which plains *more blest*, or verdant vales bestow.
> Here rocks alone and tasteless sands are found,
> And faint and sickly winds forever howl around."

And by all such selfish, contracted and obstinate *minds* as "Novice" betrays, as well as by barren, neglected *lands*, we are reminded of the lines of Dryden:

> "Salt earth and bitter are not fit to sow,
> Nor will be tamed, or mended with the plow,
> Sweet grapes degenerate there, and fruits declined
> From their first flav'rous taste renounce their kind."

In conclusion, I will only offer a remark or two on the reference "Novice" has made to the clergy, and hope, that, if this meets the eye of the respected member of that class of our fellow-citizens who, by his candor and love of truth, was the innocent occasion of the assault, he will pardon me for, noticing, even so briefly, this part of the article before me. Can Novice be ignorant of the fact, that, to no class of men, science is a greater debtor than to the clergy? Have they not ever been the guardians of true philosophy and learning in all its various branches? In the history of Astronomy, Chemistry, indeed, every branch of Natural Philosophy, do we not find, at every step, the clergy giving their valued contributions? It is a great error to suppose that a clergyman impairs his usefulness, or acts inconsistent with his position, in serving the interests of science.

Just as I am reading the complaint of "Novice," I receive from a friend a copy of "Com. Perry's Expedition to Japan," which contains numerous valuable scientific observations, by that ripe scholar, Chaplain Jones. The good Commodore, too, in selecting a suitable person to compile the work, from the materials furnished by himself and officers, chose a clergyman, (Dr. Hanks, of New York.) This is but one of the thousands of instances occurring every day, in which the members of the sacred profession are applied to to aid in the cause of secular learning; and their labors are appreciated by every civilized government as well as every private citizen, who know anything of the means by which the arts and sciences have been advanced to their present state of perfection. A. J. B.

*Baltimore.*

